# GSK3787-Loaded Poly(Ester Amide) Particles for Intra-Articular Drug Delivery

**DOI:** 10.3390/polym12040736

**Published:** 2020-03-26

**Authors:** Ian J. Villamagna, Danielle M. McRae, Aneta Borecki, Xueli Mei, François Lagugné-Labarthet, Frank Beier, Elizabeth R. Gillies

**Affiliations:** 1 School of Biomedical Engineering, The University of Western Ontario, London, ON N6A 5B9, Canada; ivillama@uwo.ca; 2 Bone and Joint Institute, The University of Western Ontario, London, ON N6A 5B9, Canada; flagugne@uwo.ca (F.L.-L.); fbeier@uwo.ca (F.B.); 3 Department of Chemistry, The University of Western Ontario, London, ON N6A 5B7, Canada; dmcrae9@uwo.ca (D.M.M.); aurbania@uwo.ca (A.B.); xmei29@uwo.ca (X.M.); 4 Department of Physiology and Pharmacology, The University of Western Ontario, London, ON N6A 3B7, Canada; 5 Department of Chemical and Biochemical Engineering, The University of Western Ontario, London, ON N6A 5B9, Canada

**Keywords:** osteoarthritis, drug delivery, particles, poly(ester amide), GSK3787, atomic force microscopy

## Abstract

Osteoarthritis (OA) is a debilitating joint disorder affecting more than 240 million people. There is no disease modifying therapeutic, and drugs that are used to alleviate OA symptoms result in side effects. Recent research indicates that inhibition of peroxisome proliferator-activated receptor δ (PPARδ) in cartilage may attenuate the development or progression of OA. PPARδ antagonists such as GSK3787 exist, but would benefit from delivery to joints to avoid side effects. Described here is the loading of GSK3787 into poly(ester amide) (PEA) particles. The particles contained 8 wt.% drug and had mean diameters of about 600 nm. Differential scanning calorimetry indicated the drug was in crystalline domains in the particles. Atomic force microscopy was used to measure the Young’s moduli of individual particles as 2.8 MPa. In vitro drug release studies showed 11% GSK3787 was released over 30 days. Studies in immature murine articular cartilage (IMAC) cells indicated low toxicity from the drug, empty particles, and drug-loaded particles and that the particles were not taken up by the cells. Ex vivo studies on murine joints showed that the particles could be injected into the joint space and resided there for at least 7 days. Overall, these results indicate that GSK3787-loaded PEA particles warrant further investigation as a delivery system for potential OA therapy.

## 1. Introduction

Osteoarthritis (OA) is the most common joint disorder worldwide and is a leading cause of chronic pain and disability [1,2]. More than 240 million people worldwide suffer from OA, at a cost between 1% and 2.5% of gross domestic product in developed countries [3]. The disease is multi-faceted, affecting numerous tissues within the joint, including cartilage, bone, and synovium. Exercise has been demonstrated to safely reduce pain and improve physical function in OA patients [4,5]. Nonsteroidal anti-inflammatory drugs (NSAIDs) can also be used, but can lead to cardiovascular [6] and gastrointestinal complications [7]. Next stage options include intra-articular injections of corticosteroids [8]. Unfortunately, none of the above treatments alter the progression of the disease [9]. Joint replacement therapy can be used for end-stage disease, but is limited by risks of infection, the potential for implant failure, and altered biomechanics which can lead to degenerative changes in other parts of the body [10,11]. Thus, improved treatments that are capable of slowing or halting OA progression are urgently needed.

In an effort to develop disease modifying treatments for OA, a greater emphasis has been placed on understanding the molecular mechanisms involved in OA [12]. A number of targets have been identified. For example, inflammatory modulators such as interleukins, [13] or the NF-κB pathway [14] have been identified as potential targets. Ion channels, such as TRPV1 [15] or voltage gated sodium channels, which are associated with pain, have also been investigated [16]. Recent research showed that activation of peroxisome proliferator activator receptor (PPAR) δ resulted in the degradation of cartilage tissue in an explant culture model [17]. In addition, cartilage specific PPARδ knockout mice were protected from post-traumatic OA (PTOA) following a destabilizing medial meniscus surgery. PPARδ antagonists have been previously developed. For example, GSK3787 was shown to have high selectivity for PPARδ [18]. However, PPARδ has important roles throughout the body, particularly in glucose and lipid metabolism [19,20,21], so the systemic administration of GSK3787 to treat OA would likely not be feasible due to the high risk of side effects.

Localized delivery of drugs into joints through intra-articular injection is recognized as a promising approach for the administration of OA therapeutics as it allows the drug to be delivered in the appropriate dose to the target tissue, while minimizing systemic exposure, and therefore potential side effects [22]. However, free drugs that are injected into the joint are subject to rapid clearance by lymphatic drainage within hours, thereby limiting their ability to achieve therapeutic effects [23]. Drug delivery systems provide an opportunity to incorporate therapeutics into a material that can provide sustained release into the joint [24]. A number of drug delivery systems for intra-articular injection have been developed, including liposomes [25], particles [26,27,28], hydrogels [29,30,31], and dendrimers [32]. Particles in particular have been shown to afford prolonged release in the joint over a period of months. For example, microparticles composed of poly(lactic-*co*-glycolic acid)(PLGA) encapsulating the corticosteroid triamcinolone were recently approved by the United States Food and Drug Administration [33].

Poly(ester amide)s (PEAs) are an alternative class of biodegradable polymers to polyesters. They have tunable thermal and mechanical properties [34], and often undergo surface erosion rather than bulk degradation, enabling controlled drug release and reduced concentrations of potentially inflammatory acidic species upon degradation [35,36]. Furthermore, PEAs have been shown to be well tolerated in joints [27,28], and in other in vitro [37,38], and in vivo [39] applications. For example, PEAs have been used as cell scaffolds for tissue regeneration and were found to support cell adhesion and proliferation [40,41]. They have also been explored for their ability to encapsulate and release cell growth factors and bactericides [42,43]. PEA particles loaded with celecoxib were shown to release the drug in response to inflammation and were well tolerated in a rat model [27]. They were also explored for the controlled release of triamcinolone [44]. We recently reported the preparation and study of celecoxib-loaded PEA particles and found that minor changes in PEA chemical structure led to large differences in the release rate of the drug [28]. The particles were also well tolerated in an ovine model. However, as noted above, the delivery of NSAIDs or corticosteroids would not lead to disease-modifying effects, so it is of interest to develop delivery systems that will enable the study of potential disease-modifying therapies.

Here, we describe the incorporation of the PPARδ antagonist GSK3787 into PEA particles. To the best of our knowledge, this is the first reported delivery system for GSK3787. Like our previously reported celecoxib-loaded PEA particles [28], the GSK3787-loaded particles were prepared through an emulsification–evaporation method but modifications to the particle preparation procedure and drug loading were required to successfully obtain particles. The particles were characterized by scanning electron microscopy (SEM), dynamic light scattering (DLS), and thermal analysis, and the release rate of GSK3787 in vitro was evaluated. Thermal analysis revealed differences in how GSK3787 was incorporated into the particles compared to celecoxib. In addition, unlike in our previous report, here we also characterized the mechanical properties of individual particles by atomic force microscopy (AFM) and examined the toxicity of the particles to primary immature murine articular cartilage (IMAC) cells. Furthermore, confocal microscopy was performed to examine the interactions between the particles and cells and a tissue explant model was used to assess the injectability of the particles and their retention in the joint.

## 2. Materials and Methods

### 2.1. General Materials

**Figure 1:**
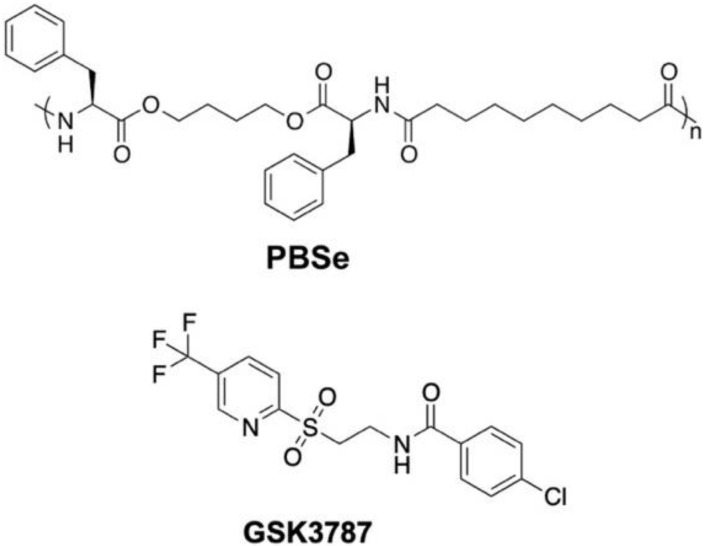
Chemical structures of the poly(ester amide) (PBSe) and GSK3787.

The PEA used in this work was composed of phenylalanine, butanediol, and sebacic acid (PBSe, Figure 1), and was synthesized and characterized as previously reported (Figures S1 and S2) [37]. For this study, the batch of polymer used had a number average molar mass (*M_n_*) of 30 kg/mol, and a dispersity (*Đ*) of 1.9. GSK3787 (98%) was purchased from Ontario Chemicals (Guelph, ON, Canada). Immunomount with 4’,6-diamino-2-phenylindole (DAPI), and CHCl_3_ (99.8%) were purchased from Fisher Scientific (Oakville, ON, Canada). Dimethyl sulfoxide (DMSO) (reagent grade), CH_2_Cl_2_ (glass distilled), DMF (glass distilled), and high-performance liquid chromatography (HPLC) grade acetonitrile (99.8%) were purchased from Caledon (Halton Hills, ON, Canada). Poly(vinyl alcohol) (PVA) 8–88 (excipient grade), 87–89% hydrolyzed was from Merck. 3-(4,5-Dimethylthiazol-2-yl)-2,5-diphenyl tetrazolium bromide (MTT, 98%), LiBr (ReagentPlus grade), and concentrated phosphate buffered saline (10×, Bioperformance grade) were purchased from Millipore-Sigma (Oakville, ON, Canada). Chemicals were used without further purification unless otherwise specified. Concentrated PBS was mixed with deionized (DI) water from a MilliQ system, to create 1× PBS, pH 7.4.

### 2.2. General Methods

Molar masses were determined by size exclusion chromatography (SEC) at a flow rate of 1 mL/min in DMF with 10 mM LiBr and 1% (*v/v*) NEt_3_ at 85 °C using a Waters 515 HPLC pump and Waters Temperature Control Module II equipped with a Wyatt Optilab T-rEX refractometer and two Plgel 5 μm mixed-D (300 mm × 7.5 mm) columns from Polymer Laboratories by Varian connected in series. The calibration was performed using poly(methyl methacrylate standards) (PMMA) standards. DLS was performed with a Zetasizer Nano ZS instrument from Malvern Instruments at 24.5 °C. The Z-average diameter and polydispersity index (PDI) for each type of particle were measured on triplicate particle preparations. Differential scanning calorimetry (DSC) was performed on a Q2000 from TA instruments (New Castle, DE, USA). The heating/cooling rate was 10 °C/min from 0 to +200 °C, and the data were obtained from the second heating cycle. Statistical analyses were performed by ANOVA tests (Microsoft Excel, 2016) with alpha set at 0.05, followed by a Bonferroni post-hoc analysis, when applicable. Animal work was performed in compliance with the guidelines of The Canadian Council on Animal Care guidelines (University of Western Ontario Protocol 2019-035).

### 2.3. Preparation of GSK3787-Loaded Particles (PBSe-GSK3787)

Particles loaded with the PPARδ antagonist, denoted as PBSe-GSK3787, were prepared through an oil-in-water emulsification evaporation method. The dispersed phase of the emulsion was made by dissolving 400 mg of PBSe in 200 mL of a 50:50 mixture of CHCl_3_:CH_2_Cl_2_. Then, 37.5 mg of GSK3787 was added to the dispersed phase simultaneously and was dissolved completely by stirring. The continuous phase was prepared by dissolving 5.0 g of PVA in 1.0 L of deionized (DI) water, in a 5 L beaker. The emulsion was formed by slowly pouring the dispersed phase into the continuous phase, while mixing vigorously using a Waring Commercial immersion blender, set to low (~9000 rpm). The solution was continuously mixed at 9000 rpm for an additional 2 min. The resultant emulsion was immediately transferred to a 1 L beaker ensuring that the liquid filled the beaker entirely, before being covered with aluminum foil, and perforated with five holes to slow the evaporation rate. The organic solvent was evaporated under constant stirring in a fume hood for 24 h. The emulsion was then transferred to 50 mL centrifuge tubes, which were spun at 2800 g for 10 min. Solid particles sedimented at the bottom of centrifuge tubes, and the aqueous layer was discarded. Particles in the tubes were resuspended in 50 mL of DI water, and were spun again for 10 min at 2800 g to wash the particles. After removing the aqueous layer, the particles were collected by resuspending the contents of each centrifuge tube in 5 mL of DI water. Fractions from different centrifuge tubes were combined and frozen overnight at −20 °C before being lyophilized, affording a ~60% yield of particles. Note that higher yields can be obtained by centrifugation at higher force, but then it is more difficult to redisperse the particles afterwards. The dried samples were kept refrigerated at 4 °C until use.

### 2.4. Preparation of Non-Drug-Loaded Particles (PBSe-NDL)

Particles without drug were prepared by the same method as for PBSe-GSK3787 except that no drug was added to the dispersed phase. The particles were recovered in ~60% yield.

### 2.5. Preparation of Dye-Labeld Particles

When required for microscopic and stereoscopic examination, particles with dyes loaded into them were prepared by the same method as for PBSe-GSK3787, with the addition of either 5 mg of Nile red (PBSe-GSK3787-NR particles) or 5 mg of IR-780 (PBSe-GSK3787-IR particles) into the dispersed phase of the emulsion instead of GSK3787. The particles were recovered in ~60% yield.

### 2.6. Scanning Electron Microscopy

SEM was performed in the University of Western Ontario’s Nanofabrication Facility using a Zeiss LEO 1530 instrument, operating at 2.0 kV and a working distance of 6 mm. Lyophilized samples of particles were mounted to stubs covered in carbon tape and coated with a 10 nm layer of Osmium, using an SPI Supplies, OC-60A plasma coater. Micrographs of the particles were taken, and images were produced to measure the size of particles. Particles in three different images and three representative sections (~30 × 30 μm^2^) per image were measured to calculate the average diameters ± standard deviation.

### 2.7. Measurement of Drug Loading and Encapsulation Efficiency

Ten milligrams of PBSe-GSK3787 particles were accurately weighed. The particles were then completely dissolved in 1 mL of dimethyl sulfoxide (DMSO). Then, 20 μL of the DMSO was taken and added to 980 μL of the high performance liquid chromatography (HPLC) mobile phase, 40:60 acetonitrile:DI water. Samples were filtered with 0.2 μm membrane filters prior to injection. HPLC analysis was then performed using an instrument equipped with a Waters Separations Module 2695, a Kinetex C18 5 μm (4.6 mm diameter × 100 mm length) column connected to a C18 guard column, and a Photodiode Array (PDA) Detector (Waters 2998). The PDA detector was used to monitor GSK3787 absorbance at 238 nm. An isocratic eluent method with acetonitrile and DI water (40:60) was used with a flow rate of 1 mL/min. The retention time of GSK3787 was 2.5 min. The calibration curve was obtained by spiking the mobile phase with known concentrations of GSK3787 to form the following standard solutions: 100, 50, 25, 10, 5, and 1 μg/mL GSK3787. All samples were filtered through 0.2 μm membrane filters, and 100 μL was injected using the instrument method described above. Three different particle preparations were used to evaluate drug loading and encapsulation efficiency, and each injection was performed in duplicate. Drug loading and encapsulation efficiency were calculated according to Equations (1) and (2).

(1) $$\%\;Drug\;Loading=\left(\frac{Mass\;of\;drug\;encapsulated\;in\;particles}{Total\;mass\;of\;particles}\right)\times 100$$

(2) $$\%\;Encapsulation\;Efficiency=\left(\frac{Actual\;GSK3787:PEA\;mass\;ratio}{Theoretical\;GSK3787:PEA\;mass\;ratio}\right)\times 100$$

### 2.8. Atomic Force Microscopy

Particles were resuspended in PBS, and deposited on glass coverslips, dropwise. After allowing liquid to evaporate at ambient temperature overnight, the samples were used for AFM imaging and mechanical testing. AFM measurements were carried out using a BioScope Catalyst AFM (Bruker) mounted on an inverted microscope (LSM 510, Zeiss, Göttingen, Germany). For indentation measurements, samples were immersed in water and heated to 37 °C, using the BioScope II Heater Stage and Veeco/LakeShore 331S Temperature Controller. Pyramidal silicon nitride MSCT cantilevers (Bruker) with a nominal spring constant of 0.1 N/m were used for contact mode imaging and indentation measurements. Determination of the spring constant of all cantilevers was carried out using the thermal noise method [45]. Images were recorded in air at a line rate of 1 Hz. For indentation measurements, the ‘point and shoot’ mode of the BioScope software was used. After hydration of the sample, an AFM image of a nanoparticle was acquired. A grid of 10 × 10 points was placed on the nanoparticle surface, and a force indentation curve was recorded at each point at a force trigger of 5 nN. At each indentation position, the Young’s modulus was determined by fitting a Hertz model (cone indenter) to the approach curve using AtomicJ [46]. One hundred different points on each of eight individual PBSe-GSK3787 particles and seven PBSe-NDL particles were used for measurements. Outliers from the data set were removed using a 1.5× IQR statistical method. Moduli were recorded as the mean ± standard deviation.

### 2.9. In Vitro Release of GSK3787

Fifty milligrams of PBSe-GSK3787 particles were resuspended in 1 mL of pH 7.4 PBS containing 2 wt.% of polysorbate 80 (sink solution) to facilitate the dissolution of the released drug. The particle suspension was then added into a Float-A-Lyzer dialysis cassette with a molecular weight cut-off of 10 kDa. Free (non-encapsulated) GSK3787 (50 mg/cassette) was also studied to ensure that the release of drug from the particles was not rate-limited by drug dissolution or transport across the dialysis membrane. Samples were placed in sealed containers with 3 mL of sink solution. All 3 mL of the sink solution was removed every 5 days for 30 days total and replaced with fresh solution. The concentration of drug in the sink solution was analyzed as using the HPLC method described above for the determination of the drug loading/encapsulation efficiency. Three replicates were studied for each of PBSe-GSK3787 and free drug and every HPLC injection was performed in duplicate. Release was calculated as the cumulative percentage of drug in the sink solution as compared to the total drug in the sample and is reported as the mean ± standard deviation.

### 2.10. Primary Chondrocyte Harvest and Culture

IMAC cells were harvested from 5-day-old CD-1 mouse pups, as previously described [47]. Briefly, pups were sacrificed and fixated to dissection plates. Cartilage was removed from the femoral heads, femoral condyles, and tibial plateaus. The tissue was then subjected to 1 h (3 mg/mL) followed by 24 h (0.5 mg/mL) incubations in Collagenase D diluted in Dulbecco’s Modified Eagles Medium supplemented with 2 mM L-glutamine, 50 U/mL penicillin, and 0.05 mg/mL streptomycin at 37 °C under 5% CO_2_. The tissue fragments were then agitated by pipetting and passed through a 50 μm cell strainer. Cells were isolated by centrifugation for 10 min at 1300 g, allowing the formation of a pellet. The pellet was washed in PBS buffer 2 times, and then resuspended in fresh media. Cells were counted by combining 40 μL of the cell suspension in media with 40 μL of trypan blue, mixing by pipetting, addition of 10 μL of the trypan blue/cell suspension to a cell counter plate, and analyses on a Bio-Rad TC20 Automated cell counter. Cells were seeded in 96-well treatment plates at a density of 5000 cells/well, in 12-well plates at a density of 3.0 × 10^5^ cells/well, or in 24-well plates at a density of 2.5 × 10^5^ cells/well and were allowed to grow to confluency for 7 days, with the media being replaced every 48 h.

### 2.11. Cytotoxicity of GSK3787 to IMAC Cells

GSK3787 was dissolved in DMSO at a concentration of 10 mg/mL and was added to cell culture media to afford concentrations of 10, 20, 30, 40, 50, 60, 70, 80, 90, and 100 μM. To each cell-containing well of a 96 well plate, 110 μL of treatment media was added, and allowed to incubate with the cells for 48 h. Cells receiving media without drug served as negative controls, and cells receiving sodium dodecyl sulfate at a concentration of 1 mg/mL served as positive controls for cell death. The media was then aspirated and replaced with 110 μL of media containing 5.0 mg/mL of MTT reagent, then the cells were incubated for 4 h. The MTT containing media was aspirated and 50 μL of DMSO was added to each well to solubilize the resulting purple crystals. The plate was then placed in a plate reader (Tecan Infinite M1000 Pro, Perkin Elmer Corporation, Waltham, MA, USA) and the absorbance at 540 nm was measured to quantify the relative metabolic activities of the cells. Four biological replicates were performed, as well as six technical replicates per plate.

### 2.12. Cytotoxicity of PBSe-GSK3787 and PBSe-NDL Particles to IMAC Cells

PBSe-GSK3787 or PBSe-NDL particles were resuspended in cell culture media to afford concentrations of 5, 10, 25, 50, 100, 150, 250, 500, 750, and 1000 μg/mL. The suspensions were sterilized by placing them under the UV light of the cell culture hood for 1 h. The MTT assay was then performed as described above for GSK3787.

### 2.13. Brightfield Imaging of IMAC Cells Treated with PBSe-GSK3787 Particles

PBSe-GSK3787 particles were resuspended in cell media at concentrations of 0, 25, 50, 100, 150, 250, 500, 750, and 1000 μg/mL, and then sterilized under the UV light of the cell culture hood for 1 h. Then, 2 mL of suspension was added to the cell-containing wells of a 12 well plate and incubated for 48 h. Cells were imaged after 48 h of incubation with particles under bright field mode using a Biotek Cytation 5 microscope at 20× magnification.

### 2.14. Confocal Microscopy of IMAC Cells Treated with PBSe-GSK3787 Particles

PBSe-GSK3787-NR particles were resuspended in culture media at a concentration of 100 μg/mL, then sterilized under the UV light of the cell culture hood for 1 h. Then, 1 mL of the particle containing media was added to each cell containing well in the 24 well plate, and then the cells were incubated for 48 h. The media was then aspirated, and the cells were washed 3 times with PBS before being fixed with a 4 wt.% paraformaldehyde (PFA) solution for 10 min at room temperature. After washing with PBS, 1 wt.% Triton X-100 was added and cells were incubated for 10 min at room temperature. Cells were washed with PBS again before adding a 1% bovine serum albumin (BSA) solution and incubating at room temperature for 30 min. AlexaFluor 488 Phalloidin stain was added to PBS at a concentration of 10 μg/mL, then 1 mL of the PBS containing AlexaFluor 488 was added to cells and incubated for 10 min at room temperature. Coverslips were washed with PBS before being removed and fixed to glass slides using Immunomount with DAPI. Slides were stored in the dark until imaging. Confocal microscopy was performed using a Zeiss LSM 900 confocal microscope. A 3D rendering of confocal images was created using Oxford Instruments Imaris ×64 software.

### 2.15. Ex Vivo Intra-Articular Injection of PBSe-GSK3787 Particles

50 mg/mL suspensions of PBSe-GSK3787-IR particles were prepared, and 5 μL was drawn up into 0.5 mL veterinary insulin syringes. Four healthy, male C57BL/6 mice of various ages were sacrificed under CO_2_. 5 μL intra-articular injections of PBSe-GSK3787-IR were performed on the medial side of right hind limbs. After injection, the limbs were resected and cultured in tissue culture medium containing 500 mL of α-minimum essential media (MEM), supplemented with 25 mg of ascorbic acid, 0.108 g/mL β-glycerophosphate, 1.0 mL BSA, 1.25 mL L-glutamine, and 10,000 μg/mL pen-strep [17]. Imaging was performed using a Leica M165C stereo microscope. Images were taken after 7 days of limb culture to qualitatively assess the presence of particles in the joint, and any diffusion of the particles through surrounding tissue.

## 3. Results and Discussion

### 3.1. Preparation and Characterization of PBSe Particles

**Figure 2:**
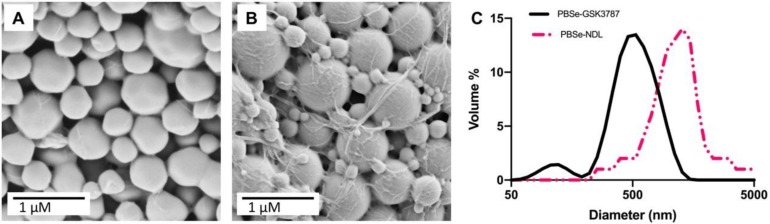
Scanning electron micrographs of particles (**A**) PBSe-GSK3787 and (**B**) PBSe-NDL (non-drug-loaded) showing their spherical morphologies and diameters in the solid state; (**C**) Representative dynamic light scattering (DLS) diameter distributions by volume % of PBSe-GSK3787 and PBSe-NDL particles showing the smaller diameters of the drug-loaded particles.

The current work employed a PEA particle delivery system based on PBSe, which was shown in our previous work to exhibit an acceptable host response in the joints of sheep [28]. Both GSK3787-loaded particles (PBSe-GSK3787) and non-drug-loaded (PBSe-NDL) control particles were prepared by an emulsification evaporation technique [48]. Initially, we investigated application of our previously developed conditions for the preparation of celecoxib-loaded PBSe particles, which involved 2 mg/mL of PBSe in CH_2_Cl_2_, 30 wt.% of celecoxib relative to PBSe, 5 mg/mL of PVA in DI water, and a 5:1 ratio of the continuous to dispersed phase [28]. At similar drug loadings of GSK3787, and even 10–15 wt.% of GSK3787, particles formed, but were contaminated with non-particle debris (Figure S3). It was suspected that the drug was disrupting the interface and/or might not exhibit high compatibility with the PEA. In contrast, clean particles were obtained at loadings of 20 mg of GSK3787 per 400 mg of PEA, and it was possible to increase this in increments of 2.5 mg up to 37.5 mg of GSK3787 per 400 mg of PEA (8.6 wt.% of GSK3787) while cleaning obtaining spherical particles (Figure 2A). In addition, it was found that the formation of particles was dependent on the evaporation rate of the organic phase of the emulsion, with a slower evaporation rate allowing for the formation of cleaner particles, without significant debris. The evaporation rates of emulsions and their effect on resultant particles has been previously studied, and the results of these studies agree with the assertion that the slower rate of evaporation used herein, is more effective for particle preparation [49,50]. To slow the evaporation rate, the dispersed phase was also changed from CH_2_Cl_2_ to 1:1 CHCl_3_:CH_2_Cl_2_. The evaporation rates of these two organic solvents has been studied in the past, with CHCl_3_ having a slower evaporation rate [51]. Furthermore, the dissolution of PEA and drug were faster in the solvent mixture than in pure CH_2_Cl_2_. Overall, these results indicate that particle preparation parameters such as drug content, solvent polarity, and solvent evaporation rate can be tuned to accommodate the loading of different drugs into a given particle delivery system. It is likely that the drug chemical structure, interfacial behavior, hydrophobicity, and compatibility with the polymer play important roles in the particle formation process.

Particles were first assessed for their morphology by SEM. The particles prepared using 8.6 wt.% GSK3787 or with no drug had approximately spherical shapes, and no major surface defects were observed (Figure 2A,B). PBSe-NDL particles had some debris as previously reported [28]. In previous work, the debris was believed to be PVA, as evidenced by the presence of a secondary T_g_ present in the DSC traces [28]. Based on SEM analysis, PBSe-GSK3787 particles had a diameter of 580 ± 290 nm, while PBSe-NDL particles had a diameter of 870 ± 74 nm (Table 1). Due to the relatively high dispersity of diameters for the PBSe-GSK3787 particles, their mean diameters were not significantly different statistically from the PBSe-NDL particles. The diameters of the particles suspended in solution and their batch-to-batch reproducibility were assessed by DLS (Figure 2C and Figure S4). The Z-average diameters were 530 ± 54 nm (PDI 0.4 ± 0.1) for PBSe-GSK3787 and 790 ± 64 nm (PDI 0.5 ± 0.1) for PBSe-NDL, indicating good reproducibility of the preparation method. In addition, the diameters obtained from SEM and DLS were quite similar. PBSe-GSK3787 particles had statistically smaller diameters than the PBSe-NDL particles based on DLS. The reduction in particle diameter and relatively high dispersity of particle diameters within a given sample might arise from the drug having a role at the solvent interface, as noted above. Overall, the particles are expected to be small enough to not induce a response through mechanical irritation, but large enough to not be rapidly cleared from the joint [52]. However, if necessary the particle size can likely be tuned in the future through tuning of the emulsion parameters.

Based on HPLC analysis (Figure S5) of dissolved particles, the drug loading of PBSe-GSK3787 particles was 8.1 ± 0.4 wt.% and the encapsulation efficiency was 94 ± 5%. The high encapsulation efficiency can be attributed to the high hydrophobicity of GSK3787, which highly favors its partition into the dispersed phase, and thus encapsulation into the particles. As noted above, the drug loading of GSK3787 was lower than what was previously obtained with celecoxib [28], due to differences in the particle preparation procedure, but this lower loading should not be a major issue. GSK3787 is known to bind to PPARδ through covalent modification of cysteine 249 on the protein, which should lead to high potency [18].

**Figure 3:**
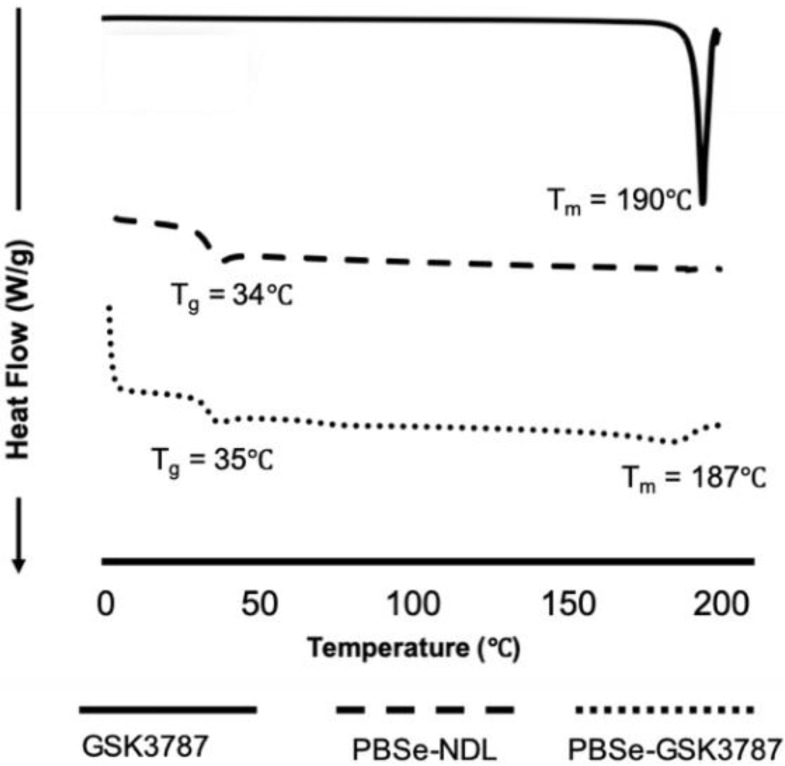
Differential scanning calorimetry (DSC) of GSK3787, PBSe-NDL, and PBSe-GSK3787. DSC shows a T_m_ for the drug and for phase separated drug in the particles. Particles both with and without drug had very similar T_g_ values, again suggesting phase separation of the drug in the particles.

DSC was performed to assess the integration of drug within the particles. Both PBSe-GSK3787 and PBSe-NDL showed similar glass transition temperatures (T_g_) of 35 and 34 °C, respectively (Figure 3). In addition, a sharp melting temperature (T_m_) was noted for the drug at 190 °C, and a broad T_m_ was observed for PBSe-GSK3787 at about 187 °C. The presence of a melting transition in the particles suggests that crystalline domains of GSK3787 were present within the particles and that the drug and polymer were phase separated. The broad transition can be attributed to domains of varying sizes. Previously, we observed homogeneous incorporation of celecoxib into PBSe particles, as evidenced by an increased T_g_ for the celecoxib-loaded particles, and no discernable T_m_, despite the drug having a melting point at 158 °C [28]. These results may explain why it was possible to incorporate celecoxib at a much higher loading of >20 wt.% compared to 8 wt.% for GSK3787.

**Figure 4:**
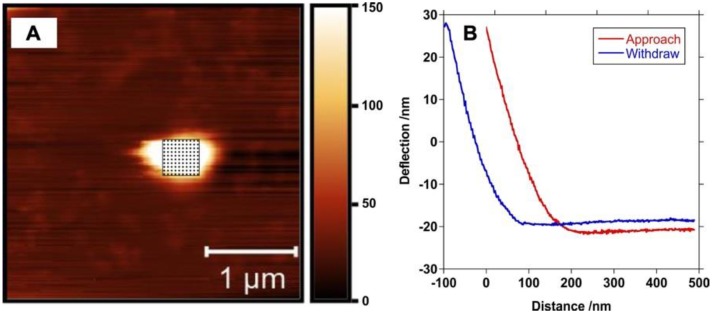
(**A**) Atomic force microscopy (AFM) image of a PBSe-GSK3787 particle showing the grid corresponding to the measurement of the modulus taken at 100 different points on a particle and (**B**) representative approach and withdrawn curves that were used to calculate the modulus.

In previous work, we characterized the Young’s moduli of bulk PEA and its blends with celecoxib by tensile testing in water at 37 °C [28]. However, the mechanical properties of the individual particles are important for their application in the joint, so in the current work AFM was used to measure the Young’s moduli of individual particles by compression with the AFM tip at 37 °C in water. The data was fit to the Hertz model (Figure 4) [53]. PBSe-GSK3787 particles had a Young’s modulus of 2.8 ± 1.0 MPa, significantly lower than the PBSe-NDL particles, which had a Young’s modulus of 8.0 ± 1.4 MPa. A reduction in modulus was also observed previously when celecoxib was incorporated into bulk PBSe and was attributed to increased plasticization of the polymer by water due to the capability of the drug to hydrogen bond to water [28]. This explanation may also apply to GSK3787 as it is also capable of hydrogen bonding. The compressive modulus of joint articular cartilage has been reported to range from 0.08 to 2 MPa, depending on the depth of tissue [54,55]. Therefore, the PBSe-GSK3787 particles have moduli of similar magnitude to cartilage. If necessary, the modulus could be further reduced by varying the PEA structure.

### 3.2. In Vitro Release of GSK3787

**Figure 5:**
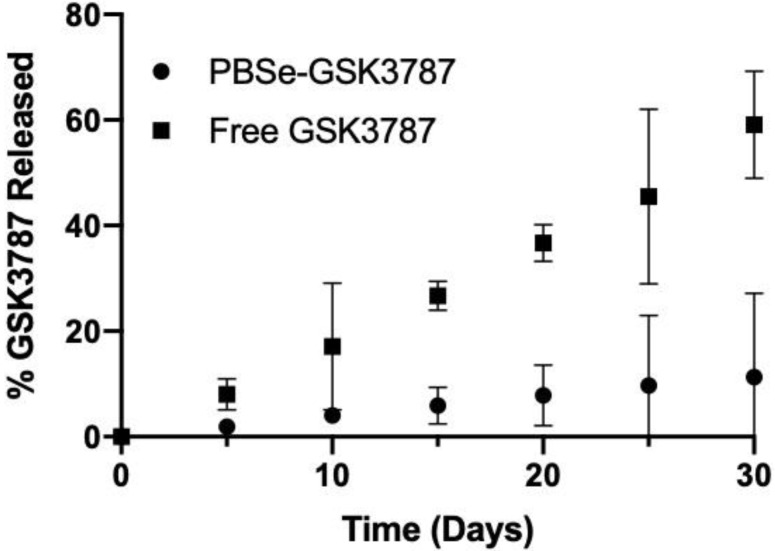
Cumulative release of GSK3787 at 37 °C in PBS containing 2 wt.% polysorbate 80. Slower release of GSK3787 was observed from PBSe-GSK3787 particles compared to the free drug. Error bars correspond to the standard deviations on triplicate samples.

The release of GSK3787 was measured by placing a 50 mg/mL suspension of PBSe-GSK3787 particles inside a dialysis cassette (1 mL) and then quantifying the concentration of drug in the external dialysate (3 mL) over time by HPLC. The experiment was performed at 37 °C in PBS containing 2 wt.% of polysorbate 80, to enhance the solubility of the drug in the release medium. The release medium was changed at each time point to ensure sink conditions. PBSe-GSK3787 exhibited a slow release of drug, with only 11% of GSK3787 released after 30 days with no burst release observed (Figure 5). The relatively high standard deviations on the cumulative release at longer time points can likely be attributed to challenges keeping the particles well dispersed in the dialysis cassette, as they tended to agglomerate during the course of the release study. The slow release of GSK3787 is a desirable feature for intra-articular drug delivery, as the clinically acceptable injection frequency is once every 3 months and it is ideal to have stable drug concentrations in the joint over this time period [56]. Although we did not study GSK3787 release for more than 30 days from the particles, previous work with celecoxib-loaded PBSe particles indicated that drug release continued at a similar rate over at least 60 days [28]. The slow drug release was attributed to slow particle degradation by a surface erosion mechanism, as intact particles were still observed by SEM at 60 days [28]. In addition, previous work involving the analysis of PBSe film and particle degradation by SEM and SEC showed that the material degraded by a surface erosion process [38,57]. This combination of work and observation of a very similar release rate for celecoxib [28] and GSK3787 over the first 30 days, strongly suggest that a similar mechanism was involved in particle degradation and GSK3787 release from PBSe-GSK3787. However, it should be noted that the actual drug release rate measured in vitro should be considered as a lower limit on the rate, as biochemical factors such as peptidases and biomechanical compression can lead to considerably faster release of drugs from delivery systems in joints [31].

To ensure that the release of GSK3787 from the delivery system was not rate-limited by simple dissolution of the drug or its transport across the dialysis membrane, a 50 mg/mL suspension of free powdered GSK3787 placed in a dialysis cassette (3 mL) was used as a control. Even at this higher drug concentration, 60% of the free GSK3787 was released into the dialysate over 30 days, showing that the drug release for PBSe-GSK3787 particles was not rate-limited by the dissolution of the drug or transport across the membrane (Figure 5). The release rate of free GSK3787 appeared remarkably zero order. It is possible that saturation of the solution with GSK3787 was reached at each time point under these conditions. In addition, dissolution of GSK3787 would be facilitated by its incorporation into micelles formed by polysorbate 80 (Figure S6) which would then transport the drug across the dialysis membrane. This could also lead to zero order kinetics as long as a depot of undissolved drug remains.

### 3.3. Cytotoxicity of GSK3787, PBSe-GSK3787, and PBSe-NDL to Primary Cell Cultures

**Figure 6:**
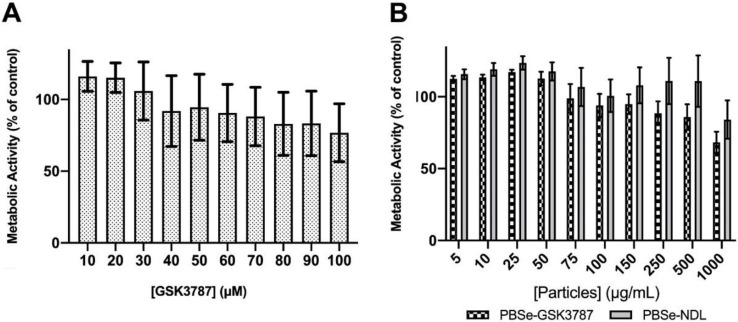
Metabolic activity of immature murine articular cartilage (IMAC) cells, as measured by the 3-(4,5-dimethylthiazol-2-yl)-2,5-diphenyl tetrazolium bromide (MTT) assay 48 h after treatment with (**A**) increasing concentrations of the PPARδ inhibitor, GSK 3787 and (**B**) PBSe-GSK3787 and PBSe-NDL particles. No significant toxicity was observed for the free drug or for PBSe-NDL. However, a trend towards higher toxicity was observed for PBSe-GSK3787 particles. Error bars correspond to standard deviations (N = 4).

IMAC cells were used in this study as they are primary cells harvested directly from immature murine pups, allowing for a cell population that is as close to cartilage as possible. Specifically, when isolated and cultured properly, IMAC cells express a number of markers that are found on chondrocytes in vivo, making them a good model for chondrocytes [58]. Free GSK3787 was first tested for cell toxicity by examining its effects on the metabolic activity using the MTT assay. No significant toxic effects were observed up to 100 μM of drug, with metabolic activities remaining greater than 80% relative to control cells not exposed to drug (Figure 6A).

**Figure 7:**
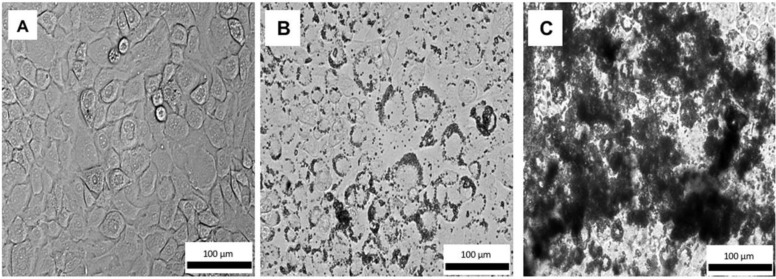
Brightfield images of live IMAC cells treated with (**A**) no particles; (**B**) 150 μg/mL of PBSe-GSK3787 particles; (**C**) 1000 μg/mL of PBSe-GSK3787 particles. The particles agglomerated and adhered to the outsides of the cell membranes. Cells treated with 150 μg/mL of particles appeared healthy, whereas the cells were almost completely coated with particles at 1000 μg/mL.

The effects of PBSe-GSK3787 and PBSe-NDL particles on IMAC cells were also evaluated using an MTT assay. There was a trend towards higher toxicity for the PBSe-GSK3787 particles, but the metabolic activities remained above 68% of the control even at 1000 μg/mL, the highest concentration tested (Figure 6B). There were no significant differences in metabolic activities between cells exposed to PBSe-GSK3787 and PBSe-NDL particles at any of the concentrations. It should be noted that at 8 wt.% drug loading, 1000 μg/mL corresponds to 80 μg/mL (~200 μM) of drug. Based on the drug release study, only a small fraction of drug should be released during the 48 h incubation, so toxic concentrations of released drug would not be expected in the assay. However, interactions of the particles with the cells may lead to high local concentrations of chemical species. Therefore, we also imaged live IMAC cells using brightfield microscopy after 48 h incubation of PBSe-GSK3787 particles with cells. The 150 μg/mL particle-treated cells remained attached to the substrate and appeared similar in shape to control cells that were not treated with particles (Figure 7 and Figure S7). At 1000 μg/mL, the cells were remarkably covered with particles. It is possible that particle coverage on the cells limited the transport of nutrients or MTT reagent to cells, reducing their apparent metabolic activity.

### 3.4. Confocal Microscopy of IMAC Cells Treated with PBSe-GSK3787-NR

**Figure 8:**
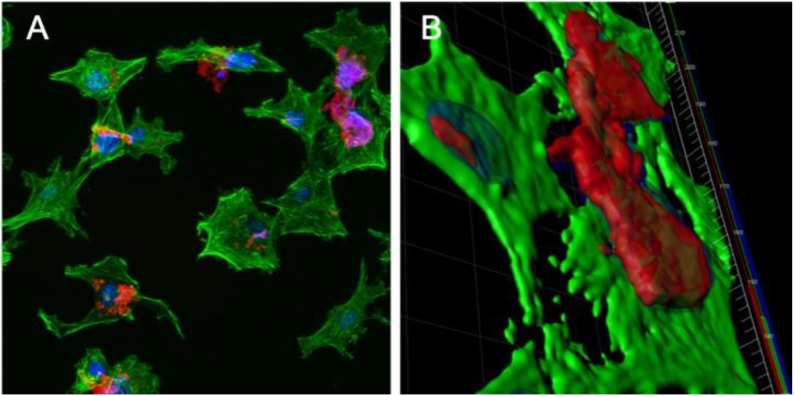
Confocal microscopy images of IMAC cells treated with 100 μg/mL Nile red-labeled PBSe-GSK3787 particles (PBSe-GSK3787-NR) particles (red) for 48 h, then stained with AlexaFluor 488 Phalloidin (green, cytoskeletons) and 4’,6-diamino-2-phenylindole (DAPI) (blue, nuclei): (**A**) 2D image showing agglomerates of particles on the cells; (**B**) 3D rendering of cells showing particles localized at the cell surface and not taken up by the cells.

Nile red-labeled PBSe-GSK3787 particles (PBSe-GSK3787-NR) were prepared to enable visualization of the particles using fluorescence confocal microscopy. We first checked whether 1.25 wt.% of dye had any impact on the particle size and found that it did not (Figure S8). IMAC cells were incubated with 100 μg/mL of PBSe-GSK3787-NR particles for 48 h, and then imaging was performed to assess how the particles interacted with the cells and whether they were taken up by the cells. The cellular actin cytoskeleton was also stained with AlexaFluor 488-Phalloidin (green) and the nuclei were stained with DAPI (Figure 8A). A 3D image rendering of the confocal images showed that the particles primarily remained at the cell surface (Figure 8B). The particles were somewhat agglomerated, and thus concentrated in certain regions rather than being uniformly distributed on the cell surfaces. However, it is likely that some particles that were initially on cells were washed away through the numerous washing steps that were associated with the staining of the cells.

### 3.5. Ex Vivo Intra-Articular Injections

**Figure 9:**
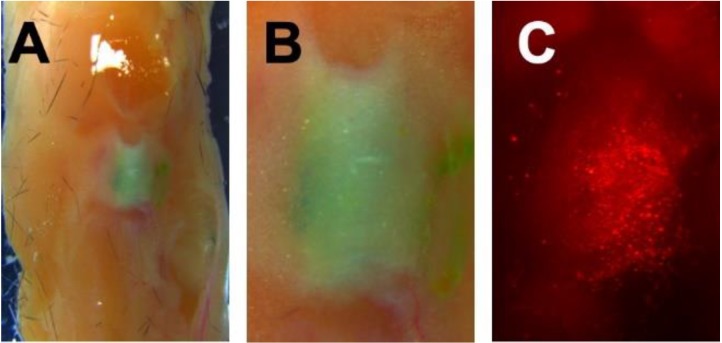
Representative knee joint explant from a C57BL/6 mouse that was injected with 5 μL of a 100 mg/mL suspension of particles with hydrophobic green dye IR780 (PBSe-GSK3787-IR) particles. Upon resection of the limbs, images were taken with a stereoscope to determine injectability and localization of particles. Images taken 7 days post injection of (**A**) Knee joint at 7.3× magnification; (**B**) Knee joint at 1.6× magnification; (**C**) Particles as visualized in the joint under fluorescence microscopy, 1.6× magnification.

For intra-articular injections, the particles were labeled with the hydrophobic green dye IR780 (PBSe-GSK3787-IR) to provide contrast against tissues in brightfield imaging as well as fluorescence at various wavelengths. Murine knee joints were obtained from C57BL/6 mice and were injected with 5 μL of a 100 mg/mL suspension of particles per joint into the intra-articular space. The joints were then resected and cultured in organ culture media for 7 d. The culture of joints has been determined previously to be a good model for the study of OA, because of its low expense, and ability of the tissue to maintain cytokine stimulation and osmotic pressure while in culture [59]. The joints were imaged to qualitatively assess the diffusion of particles away from the joint space and through the surrounding tissue. Using brightfield imaging, it was observed that distribution of particles had remained localized to the joint after 7 d, with no distinct green dye seen outside of the joint space. Fluorescence microscopy at 7 d showed that while there was particle migration through both the joint and the limb, the bulk of the injected material remained within the joint space (Figure 9). Thus, the injection into joints ex vivo allowed for a better understanding of the distribution of particles post administration, and how they behave, at least in the absence of mechanical loading.

## 4. Conclusions

PBSe particles containing the PPARδ antagonist, GSK3787, were successfully prepared by modifying our previously developed procedure. Specifically, it was important to lower the loading of drug from 30 wt.% for celecoxib to 8.6 wt.% for GSK3787 in order to achieve clean particle formation. This requirement may arise from GSK3787 acting at the interface, as supported by the formation of smaller particles in the presence of this drug, or due to incompatibility of the drug and PBSe, which was suggested by thermal analysis of the particles. Relative to particles without drug, the loading of GSK3787 into the particles lowered the Young’s modulus, bringing it closer to the natural range of articular cartilage. The particles exhibited a slow release of GSK3787 in vitro with no burst release observed. GSK3787 exhibited low toxicity to IMAC cells, as indicated by the MTT assay. The particles also exhibited low toxicity, except at the highest concentrations studied (>500 μg/mL) and this lowering of metabolic activity might be due to the high concentrations of particles localized on the cell surface, as indicated by bright field and fluorescence confocal microscopy. Knee joint explant cultures that were injected with particles showed that the particles remained mainly localized in the joint, even after 7 days of injection. Therefore, this system encapsulates and releases a potent PPARδ antagonist that cannot be delivered systemically and serves as a promising vehicle for further investigated in intra-articular drug delivery for the treatment of OA.

## Figures and Tables

**Table 1:** Physicochemical properties of PBSe-GSK3787 and PBSe-NDL particles.

Particle Composition	Z-Average Diameter (DLS) (nm)	Particle Diameter (SEM) (nm)	GSK3787 Loading (wt.%)	GSK3787 Encapsulation Efficiency (%)	Young’s Modulus (MPa)	*T*_g_ (°C)	*T*_m_ (°C)
PBSe-NDL	790 ± 64	870 ± 74	-	-	7.0 ± 1.4	34	-
PBSe-GSK3787	530 ± 54	580 ± 290	8.1 ± 0.4	94.0 ± 4.8	2.8 ± 1.0	35	187

## Supplementary Materials

The following are available online at https://www.mdpi.com/2073-4360/12/4/736/s1, Figure S1: NMR spectrum of PBSe; Figure S2: SEC trace for PBSe; Figure S3: Scanning electron micrographs of particles prepared with different amounts of GSK3787 added; Figure S4: DLS diameter distributions of the particles by intensity %; Figure S5: Representative HPLC trace of GSK3787 as measured for drug loading and encapsulation efficiency of the particles; Figure S6: DLS data for GSK3787 in the presence of polysorbate 80; Figure S7: Zoomed brightfield microscopy images; Figure S8: DLS data for particles containing Nile red or IR-780.
