# Supplementary material for: GSK3787-Loaded Poly(Ester Amide) Particles for Intra-Articular Drug Delivery
Source: Polymers (Basel). 2020 Mar 26;12(4):736. doi: 10.3390/polym12040736 (PMC7240550; doi:10.3390/polym12040736)
Supplement: Supplementary file 1 [file polymers-12-00736-s001.pdf]

Supporting information for:

GSK3787-loaded Poly(ester amide) Particles for Intra-articular Drug Delivery

Ian J. Villamagna [1,2], Danielle M. McRae [3], Aneta Borecki [3], Dawn Bryce [4], François Lagugné-Labarthet [2,3], Frank Beier [2,4], and Elizabeth R. Gillies [1,2,3,5]*

[1] School of Biomedical Engineering, The University of Western Ontario, London, Ontario, N6A 5B9, Canada
[2] Bone and Joint Institute, The University of Western Ontario, London, Ontario, N6A 5B9, Canada
[3] Department of Chemistry, The University of Western Ontario, London, Ontario, N6A 5B7, Canada
[4] Department of Physiology and Pharmacology, The University of Western Ontario, London, Ontario, N6A 3B7, Canada
[5] Department of Chemical and Biochemical Engineering, The University of Western Ontario, London, Ontario, N6A 5B9, Canada
* Correspondence: egillie@uwo.ca; Tel.: 519-661-2111 ext 80223

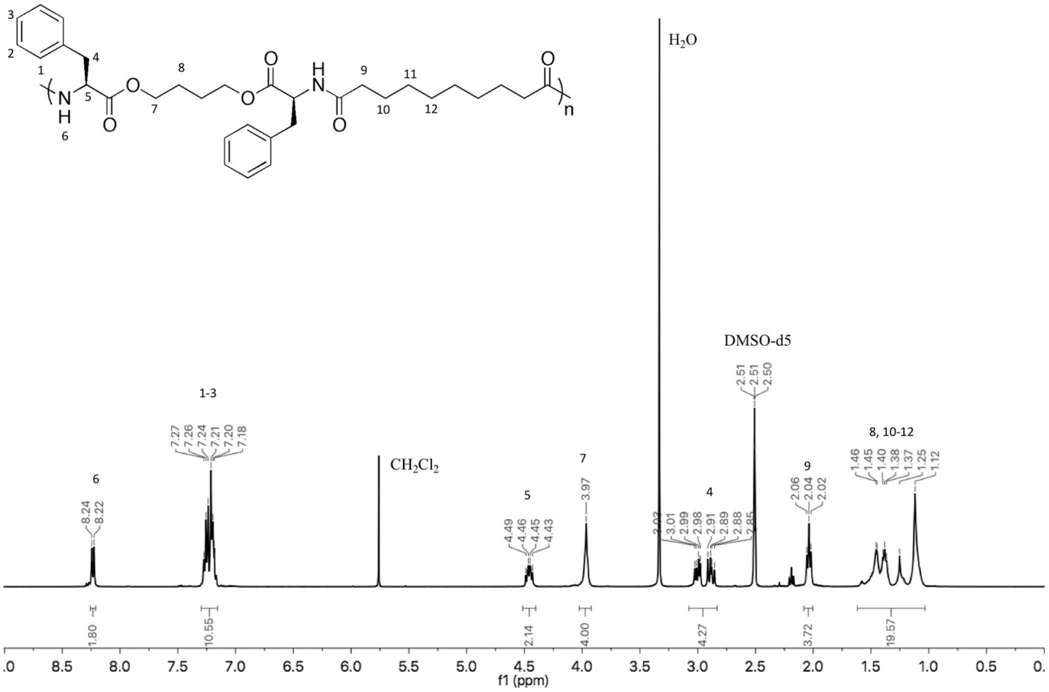

Figure S1. ^{1}H NMR spectrum of PBSe (DMSO-d_6, 400 MHz).

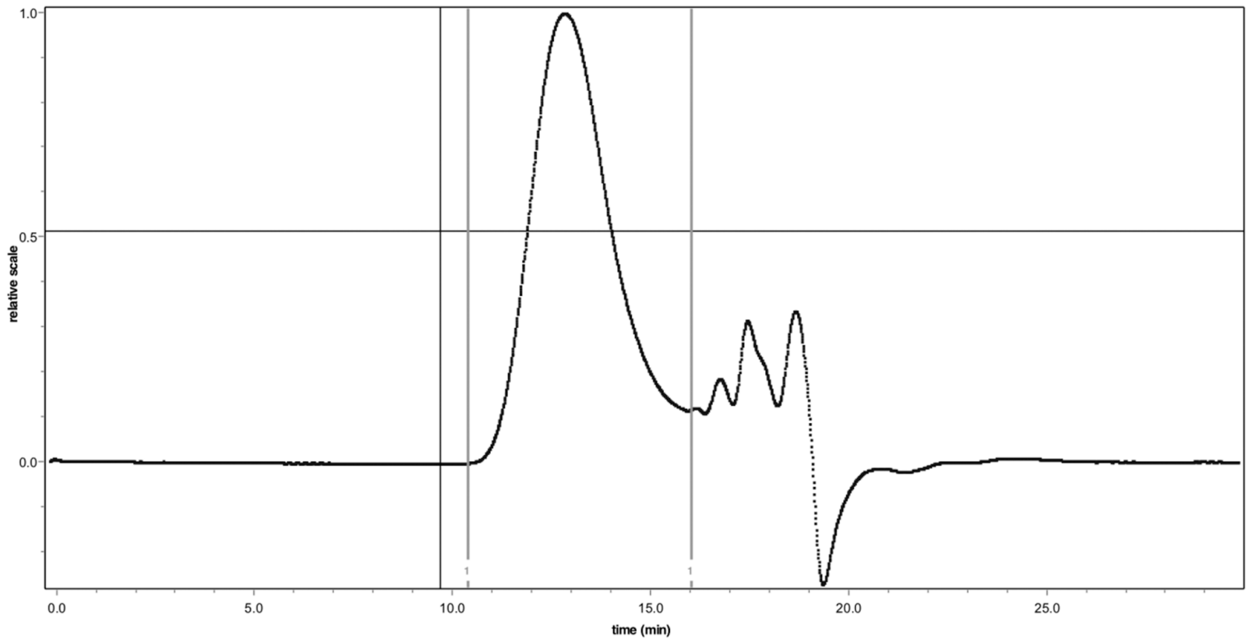

Figure S2. SEC trace (DMF eluent containing 10 mM LiBr, refractive index detection) for PBSe (M_n = 30 kg/mol and $Đ$ = 1.9).

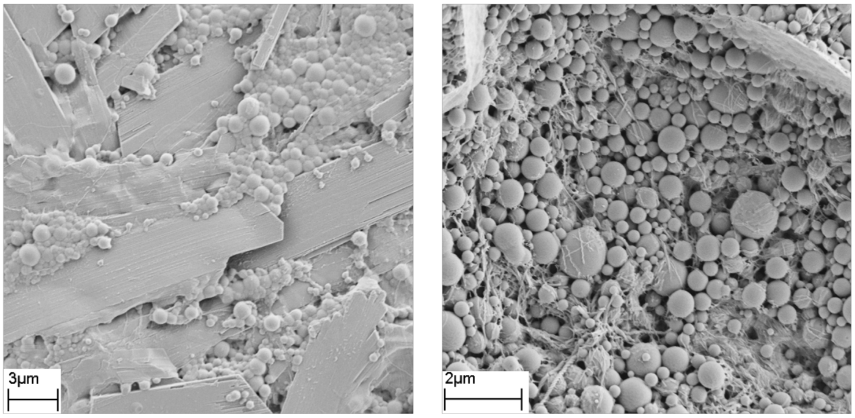

15% 10%

Figure S3. Scanning electron micrographs of particles prepared with different amounts of GSK3787 added. Particles with 15 wt% of GSK3787 added to the dispersed phase of the emulsion did form, but in small numbers and with large amounts of excess, non-particle, material (left). Particles with 10 wt% of GSK3787 did form, and were of spherical morphology and had a good size distribution, but had visible polymer remaining in the samples (right).

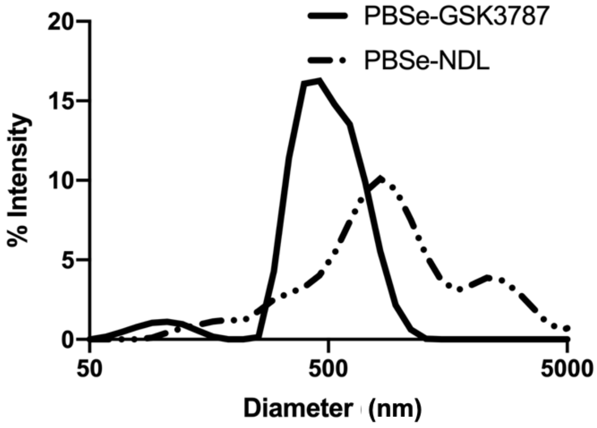

Figure S4. DLS diameter distributions by intensity % of PBSe-GSK3787 and PBSe-NDL particles showing the smaller diameters of the drug-loaded particles.

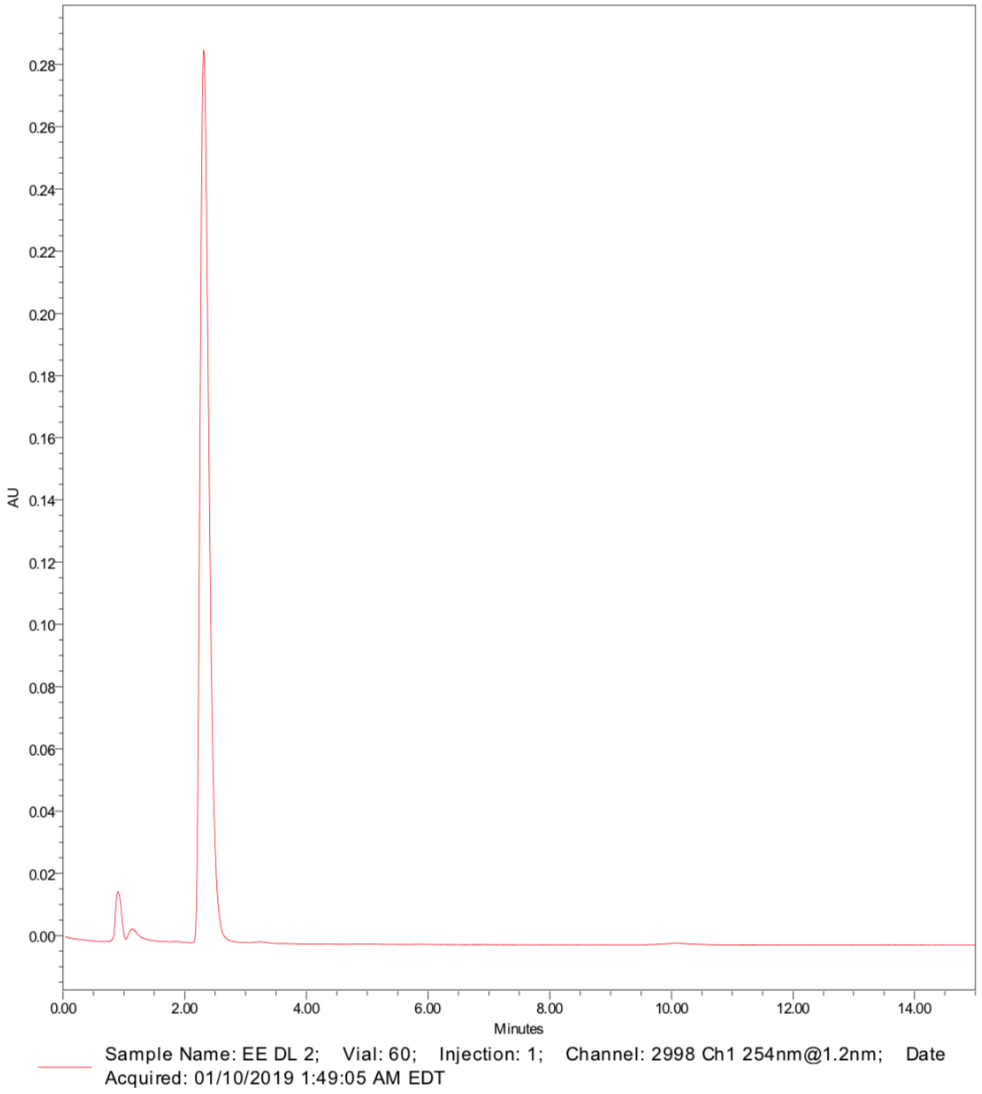

Figure S5. Representative HPLC trace of GSK3787 as measured for drug loading and encapsulation efficiency of particles.

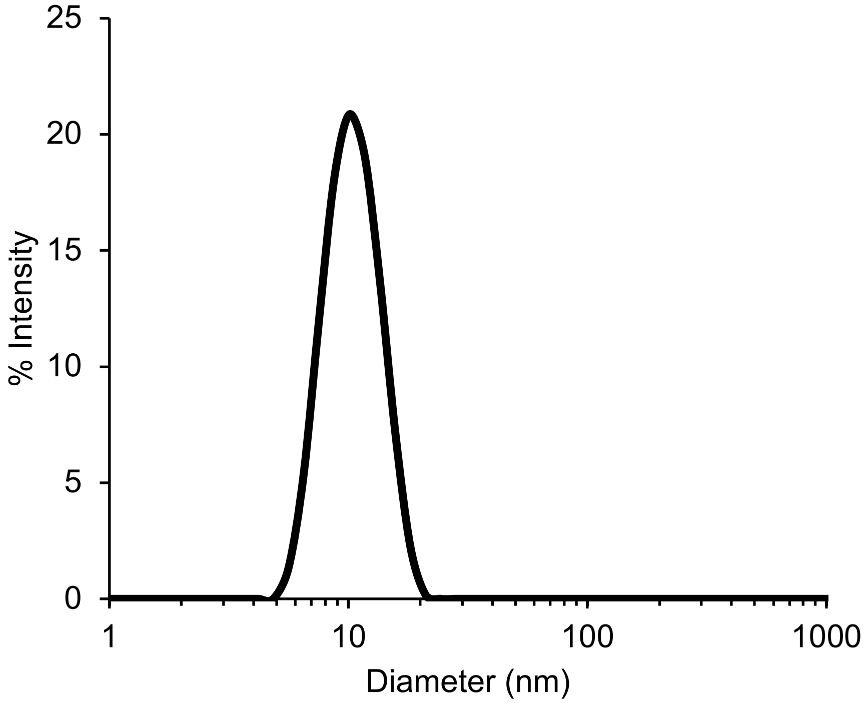

Figure S6. DLS diameter distributions by intensity % of GSK3787 (0.1 mg/mL) in a 2 wt% solution of polysorbate 80 in PBS at 37 °C. The absence of turbidity and presence of assemblies with diameters of ~10 nm suggest that the drug was incorporated into polysorbate micelles at this concentration.

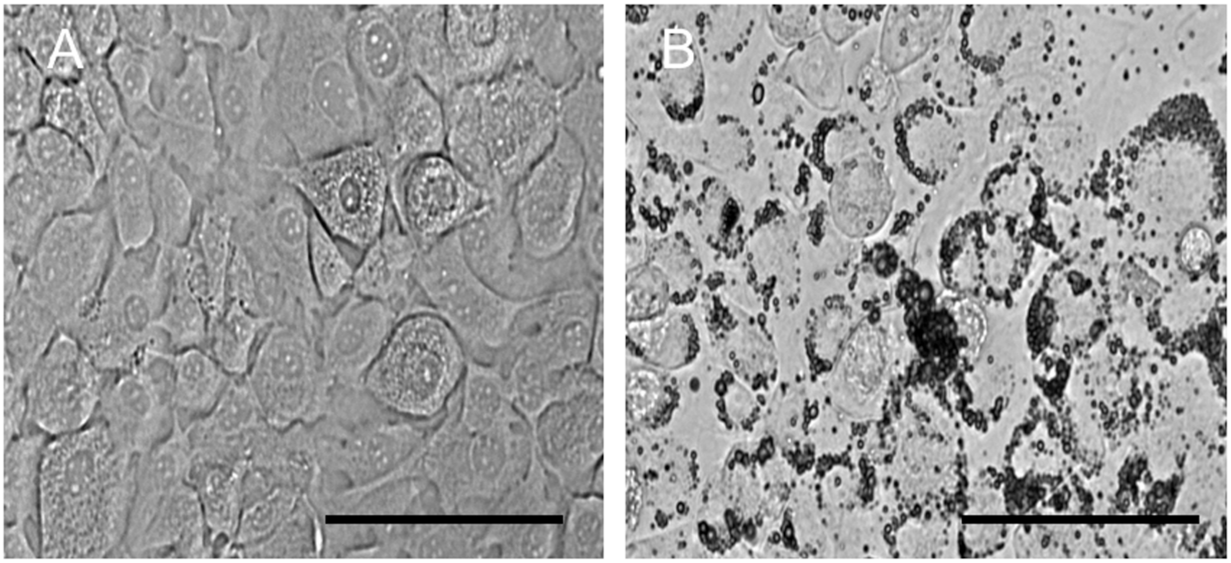

Figure S7. Zoomed brightfield images of live IMAC cells treated with A) no particles; B) 150 μg/mL of PBSe-GSK3787 particles. No noticeable changes in cell morphology were observed. Scale bar = 100 μm.

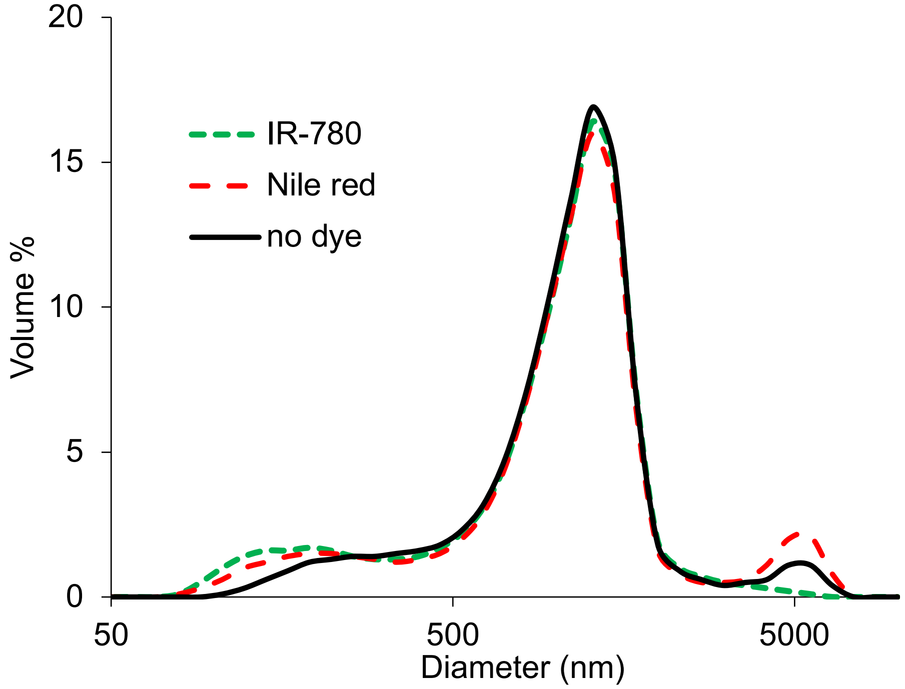

Figure S8. Representative DLS diameter distributions by volume % of PBSe-NDL particles prepared without dye, with 1.25 wt% Nile red, or with 1.25 wt% IR-780. All led to very similar size distributions.
